# Massive thymic hemorrhage and hemothorax occurring in utero

**DOI:** 10.1186/s13052-015-0196-5

**Published:** 2015-11-14

**Authors:** Giancarlo Gargano, Anna Lucia Paltrinieri, Claudio Gallo, Luciana Di Pancrazio, Maria Federica Roversi, Fabrizio Ferrari

**Affiliations:** Department of Obstetric and Pediatric, Neonatal Intensive Care Unit (NICU), Arcispedale S. Maria Nuova, Istituto Tecnologie Avanzate e Modelli Assistenziali in Oncologia-IRCCS, Viale Risorgimento n. 70, 42100 Reggio Emilia, Italy; Department of Obstetric and Pediatric, Neonatal Intensive Care Unit (NICU), University Hospital, Modena, Italy; Department of Radiology, University Hospital, Modena, Italy

**Keywords:** Thymic haemorrhage, Haemothorax, Respiratory distress, Newborn, Thymus, Haemorrhage, Mediastinal mass, Newborn infant

## Abstract

**Background:**

Thymic enlargement is a common and physiological finding in children and neonates’ X-rays, but it is usually asymptomatic. Occasionally it can cause respiratory distress. In most cases the aetiology of this expansion remains unclear and it is diagnosed as a thymic hyperplasia. True thymic hyperplasia is defined as a gland expansion, both in size and weight, while maintaining normal microscopic architecture. Often it is a diagnosis of exclusion and prognosis is good.

Thymic haemorrhage is an unusual condition related to high foetal and neonatal mortality.

**Case Presentation:**

We report a case of spontaneous massive thymic haemorrhage in a newborn developing at birth acute respiratory distress associated with severe bilateral haemothorax.

Thymic enlargement was evident after pleural evacuation and confirmed by radiographic, Computed Tomography (CT) images and Magnetic Resonance Imaging (MRI) sequences. The spontaneous resolution of this enlargement seen with CT scan and MRI sequences suggested a thymic haemorrhage; surgery was not necessary.

**Conclusion:**

Thymic haemorrhage should be considered in newborn infants with pleural effusion, mediastinal space enlargement and Respiratory Distress.

## Background

Respiratory Distress Syndrome (RDS) is a clinical presentation of many diseases in the neonatal period and is one of the most frequent causes of admission to the neonatal intensive care unit (NICU), both in term and preterm infants. The etiology of RDS in term newborn infants includes pulmonary and extra pulmonary diseases, with a pronounced prevalence of the first ones. Among the extra pulmonary causes, even if rare, it has to be mentioned a mediastinal mass, which can lead to external compression on the airways and thus lead to RDS.

Thymic enlargement is a common feature in newborn-infants chest-x-ray, but usually it is asymptomatic and the finding is absolutely accidental.

Conversely thymic haemorrhage is an extremely rare condition in the neonatal period. To our knowledge only eight cases have been published to date. The mortality is very high: two of the eight cases had a lethal outcome, other five required surgery and only one case resolved following medical treatment. The aetiology is often unknown, but different causes were suggested: birth trauma, haemorrhagic disease, coagulopathy, erythroblastosis fetalis, cyst and tumour.

Here we describe a case of thymic haemorrhage with perinatal onset, associated with bilateral haemothorax and severe respiratory distress at birth.

## Case presentation

Male infant born at 38 weeks of gestation by emergency caesarean section due to acute foetal distress, in particular finding of reduced foetal movements and reduced foetal cardiac variability on the CTG. The ultrasound scan performed on the day of delivery showed evidence of pleural and peritoneal effusion.

The pregnancy had been uneventful, normal antenatal scan and normal serology. No record of maternal drug assumption during the whole pregnancy and before delivery.

Birth weight was 3930 gr (95^th^ centile).

The obstetrical history revealed a previous full term stillbirth, followed by a physiological normal pregnancy. On the first stillborn infant standard investigations were carried out, according to our onsite protocol (genetics, infection and vascular investigations, histopathological examination and autopsy). All the results came back negative, and the stillbirth was classified as Sudden Intrauterine Unexplained Death.

At birth, the newborn presented with severe cardio-respiratory depression (Apgar 1 at 1’, 7 at 5’) requiring oro-tracheal intubation and external cardiac massage, resulted in prompt recovery of the heart rate, but prolonged need for ventilator support. The baby was then admitted to the neonatal intensive care unit and mechanical ventilation was started. The chest X-ray showed a bilateral pulmonary opacity related to pleural effusion. The chest-abdomen ultrasound revealed abundant bilateral pleural fluid and a minimum peritoneal effusion; it also showed a heterogeneous hyperechoic mass, likely to be thymic. The echocardiogram revealed the presence of a modest pericardial effusion and confirmed the mediastinal hyperechoic mass. Temporary cannulas inserted in the left and right pleura at 90 min of age confirmed a bilateral haemothorax, confirmed by the biochemical analysis of the fluid obtained.

**Fig. 1:**
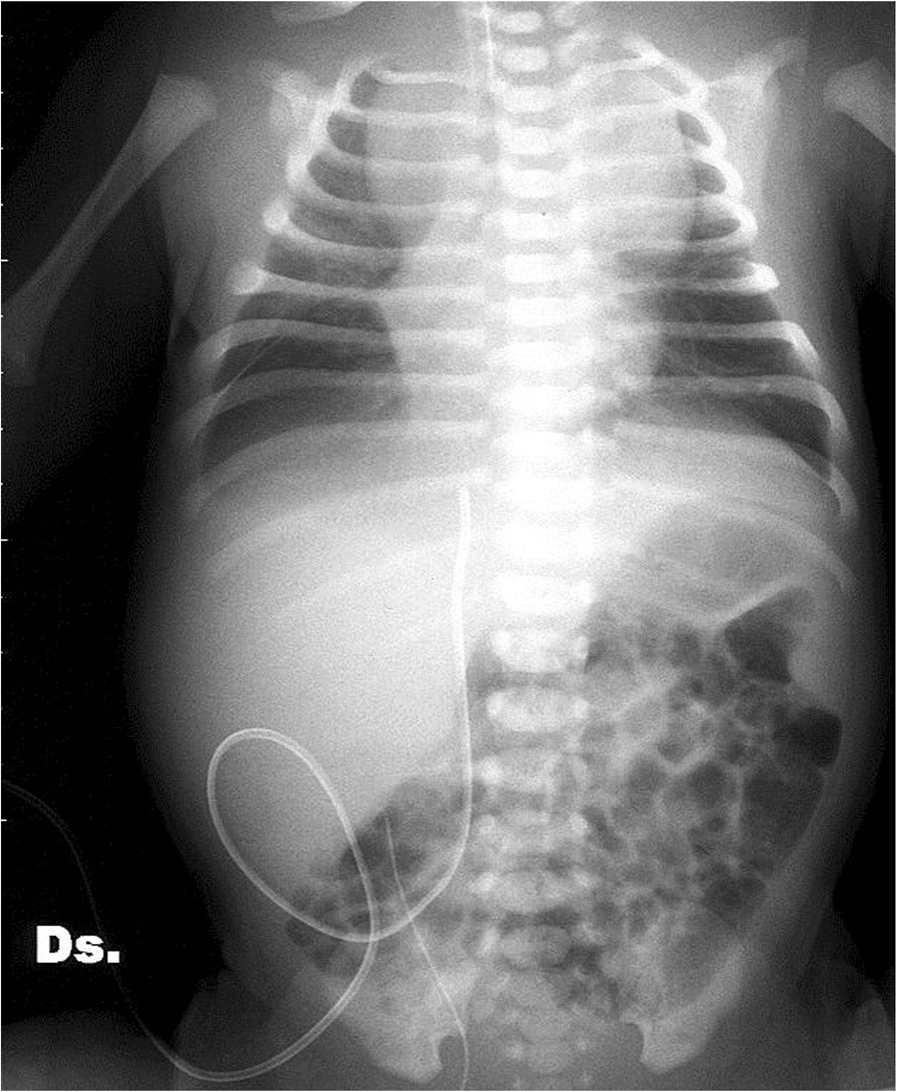
Chest X-ray on day 3: pleural effusion resolution; relevant mediastinum enlargement

**Fig. 2:**
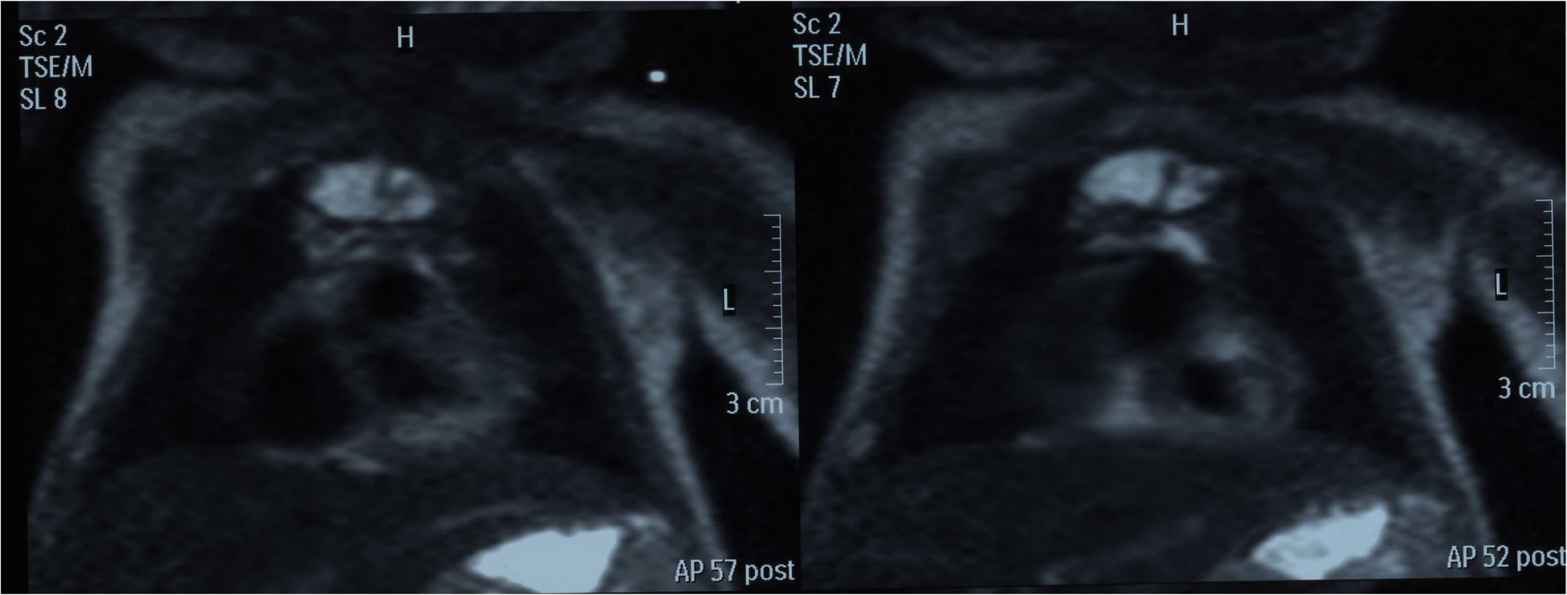
MR-sequences on day 16: thymus occupies the antero-superior mediastinum, with greatly reduced dimensions compared to the previous CT scan. Thymus appears to be homogeneous and with hyperintense signal in T1 as in T2 sequences. This aspect is suggestive of a recent bleeding. (Intera 1,5 T- Philps)

In view of this finding, bloods were performed on admission, including a coagulation screening. Severe alteration of coagulation factors (PT 102 s and aPTT>120 s with normal fibrinogen and antithrombin III) and mild anaemia (Hb 10.4 g/dl, Ht 31.7 %, erythrocytes 2.82 x 10^6^/μl, PLT 282.0 x 10^9^/L) were found. A supplementary dose of vitamin K was given (2 mg im in total) together with fresh frozen plasma at 15 mls/kg and red packed cells transfusion at 20 mls/kg. Surgical intervention was not needed. After 24 h the coagulation normalized and so it remained subsequently. On the 2^nd^ day, the newborn was able to breathe unassisted. The radiological control revealed a reduction of the bilateral pleural effusion and an enlargement of the superior mediastinal limbus (Fig. 1). The ultrasound disclosed an enlarged thymus in the antero-superior mediastinal space (6 cm of longitudinal diameter, 3 cm of antero-posterior diameter) with irregular limits and non-homogeneous structure. The chest CT, performed on day four of life, confirmed the presence of an enlarged thymus, mainly developed in the left paramedian zone. The trachea appeared to be dislocated to the right. In non-contrast CT- sequences, the thymus appeared with hyperdensity areas, as from a recent bleeding. The mass still looked non-homogeneous after the contrast enhancement. After nearly 10 days, a chest MRI was performed (Fig. 2). It revealed a thymus of very reduced dimension, with a homogeneous structure and hyperintense signal in both T1 and T2 sequences, compatible with the consequences of a subacute haemorrhage. Signal hyperintensity in T2w moreover than in T1w sequences is due to extracellular methemoglobin concentration caused by erythrocytes lysis, which occurs after about one week of acute bleeding and gradually becomes more intense.

Other investigations to identify possible antenatal infections (TORCH, Epstein Barr virus serology, Parvovirus B19, Adenovirus, Enterovirus, Cytomegalovirus) were negative. A coagulation study on maternal blood didn’t reveal any abnormality. The main tumour markers (chorionic gonadotropin, alpha-fetoprotein, enolase, vanilmandelic acid) were negative. An accurate history was taken from parents about themselves and their families, but no history of coagulopathy or any other hematological condition emerged.

The newborn was discharged on day 29 in good clinical condition. A chest X-ray was performed before discharge and showed a complete resolution of the mass.

At 2 years old, the child is doing well and the radiological control is negative.

## Discussion

Thymic enlargement is a common accidental finding on chest x- rays in healthy neonates and infants, but there are usually no clinical symptoms and complications.

Thymus pathologic lesions are rare in infancy and the aetiology remains often unclear.

Thymic haemorrhage in the perinatal period is an exceedingly rare and severe condition, often leading to stillbirth or to severe respiratory distress at birth. In 1971 Ribet et al. [4] reported the first known case, with a positive outcome after surgery. Then 2 cases of spontaneous thymic haemorrhage with positive outcome after surgery are described in 1974 by Woolley et al. [10]. Several years after Urvoas et al. [7] presented a case of spontaneous thymic haemorrhage in a 4-week-old boy, with good resolution after medical treatment. In the case reported by Walsh et al. [9] in 1996, the newborn showed severe neonatal respiratory distress, which led to death at a few hours of age. The diagnosis was therefore post-mortem. Subsequently, Bees et al. [1] described a neonatal thymic haemorrhage, resolved only after surgical intervention. In 2001 Saksenberg et al. [6] reported a case of intrauterine death in which the autopsy revealed a thymic haemorrhage, with simultaneous cerebral haemorrhage. More recently Eifenger et al. [3] described a case of acute life-threatening thymic bleeding from a thymic cyst ruptured into the pleural space in a 5 week old male infant, which resolved after surgery.

In Table 1 we report a summary of the cases of thymic haemorrhage reported in literature with details of type of treatment received and outcome of each case.

**Table 1:** Thymic haemorrhage in the perinatal period

Author	Case	Treatment	Outcome
Ribet M et al. 1971 [4]	Newborn	Surgical, total excision	Positive
Woolley MM et al. 1974 [10]	One newborn, one 4 weeks old infant	Surgical, total excision	Positive
Urvoas E et al. 1994 [7]	4 weeks old infant	Medical	Positive
Walsh SV et al. 1996 [9]	Newborn		Lethal
Bees NR et al. 1997 [1]	Newborn	Surgical, total excision	Positive
Saksenberg V et al. 2001 [6]	Intrauterine death at 37 weeks and 4 days		Lethal
Eifenger F et al. 2007 [3]	5 weeks old infant	Surgical, total excision	Positive

Summary of the cases reported in literature

We report a case of thymic haemorrhage with difficult diagnosis and unclear aetiology but with a positive spontaneous outcome. To our knowledge, this is the ninth case reported: two cases died, in five cases surgery was necessary and just one case resolved after medical treatment. We speculate that, in our patient, the emergency caesarean section interrupted a series of events that could have led to a stillbirth.

In our case the mediastinal space enlargement revealed itself only after the progressive haemothorax drainage. The mass location and its non-homogeneous structure were disclosed by the chest ultrasound scan and then confirmed by the CT scan. A non-homogeneous mass in the antero-superior mediastinal space, causing the dislocation of all close structures, such as esophagus, trachea and blood vessels, suggested a thymic or tumoral origin (lymphangioma). The anterior mediastinal space is the natural location of the thymus and the ultrasound and CT images confirmed it. The dishomogeneously hyperechoic aspect excluded a lymphangioma. Moreover the negativity of the main tumoral markers and the dimensional reduction at the following MRI control, excluded the tumoral mass (due to lymphangioma, lipoma or teratoma). The non-homogeneous structure of the gland, as well as the simultaneous haemothorax associated with severe coagulation abnormalities, supported the hypothesis of thymic haemorrhage. Surgical intervention was not necessary: the newborn clinical condition improved gradually, along with the radiological pattern.

Regarding the possible aetiology of the thymic haemorrhage we took several possibilities into considerations. In absence of traumatic events during pregnancy, and with a negative family history, we believe it might have been determined by a spontaneous haemorrhage, which might have caused a fast consumption of the coagulation vitamin K dependent factors that are already poor in the newborn. In our case we assume that the coagulation abnormalities were a consequence of the haemorrhagic event.

A second hypothesis is for the haemorrhage to be a direct consequence of a primitive coagulation alteration, occurred in the terminal stage of pregnancy. But this hypothesis could be excluded by the normality of the maternal coagulation and the absence of a clear pattern of haemorrhagic diathesis, which had to involve other typical locations of neonatal bleeding, such as the brain and the gastric mucosa. The fibrinogen normal value excluded a disseminated intravascular coagulation (DIC). The simultaneous haemothorax could be explained as the consequence of a visceral pleural tearing, as often is detected in operating theatres and not necessarily as a haemorrhagic event itself [2]. We took also into consideration the possibility of a Vitamin K deficiency, in view of the prolonged PT and aPTT with normal fibrinogen and platelets count. The second dose of vitamin K given after viewing the coagulation results might have interrupted further bleeding from multiple sites. We find though this hypothesis unlikely, as the infant didn’t present any bleeding involving skin or mucosae, which are common presentation in Vitamin K deficiency cases. Maternal bloods didn’t show any abnormality though and the child has been healthy in the following two years of life.

The moderate pericardial and peritoneal effusion could be due to an initial hydrops, as described by other authors [5], concomitant to the anemic condition, but it could also be related to the vascular peduncle compression by the thymic mass, with the consequent reduction of the cardiac output.

We don’t believe the cardio-pulmonary resuscitation to be a possible underlying cause of the bleeding of the hypertrophic thymus, as the baby heart rate came back very quickly, by one minute after chest compression were started and the pleural effusion was already evident before delivery.

Thymic haemorrhage, as suggested in Vestfrid publication [8], could be underestimated, because often misdiagnosed. However, we advise for this hypothesis to be considered in all newborns presenting at birth with respiratory distress, mediastinum enlargement and pleural effusion.

CT scan and MRI sequences allow better definition of mediastinum structures and can be helpful in the diagnosis process and in the bleeding timing detection.

## Conclusion

Respiratory Distress Syndrome is one of the most common presentations of neonatal diseases and it can be a manifestation of both pulmonary and extra-pulmonary illnesses. Sometimes thymic enlargement can cause respiratory failure as well.

Conversely, thymic haemorrhage is an extremely rare condition in newborns and it is related to high foetal and neonatal mortality. It requires early diagnosis and emergency therapeutic measures in order to improve the prognosis. We have to consider this hypothesis in all newborns presenting at birth with respiratory distress, mediastinum enlargement and pleural effusion.

Radiography is useful as a first assessment, but only ultrasound, CT and MRI images can allow better characterization of the disease and can help the diagnostic process and the definition of the bleeding timing.

## Consent

Written informed consent was obtained from both parents for publication of this Case report and accompanying images. A copy of the written consent is available for review by the Editor-in-Chief of this journal upon request.

## Abbrevations

CT: computed tomography
MRI: magnetic resonance imaging
RDS: respiratory distress syndrome
NICU: Neonatal Intensive Care Unit
PT: prothrombin time
PTT: partial thromboplastin time
PLT: platelets
DIC: disseminated intravascular coagulation

## References

[1] Bees NR, Richards SW, Fearne C, Drake DP, Dicks-Mireaux C (1997). Neonatal thymic hemorrhage. Br J Radiol.

[2] Day DL, Gedgaudas E (1984). Symposium on nonpulmonary aspects in chest radiology. The thymus. Radiol Clin North Am.

[3] Eifinger F, Ernestus K, Benz-Bohm G, Korber F, Hunseler C, Hekmat K, Marx A, Roth B (2007). True thymic hyperplasia associated with severe thymic cyst bleeding in a newborn: case report and review of the literature. Ann Diagn Pathol.

[4] Ribet M, Ponte C, Lequien P, Lacombe A, Gosselin B (1971). Tumeur du thymus, hémothorax et détresse respiratoire chez un nuveau-né. Presse Med.

[5] Rocha G, Fernandes P, Rocha P, Quintas C, Martins T, Proenca E (2006). Pleural effusions in the neonate. Acta Paediatr.

[6] Saksenberg V, Bauch B, Reznik S (2001). Massive acute thymic haemorrhage and cerebral haemorrhage in an intrauterine fetal death. J Clin Pathol.

[7] Urvoas E, Pariente D, Rousset A, De Victor D, Leblanc A (1994). Ultrasound diagnosis of thymic hemorrhage in an infant with late-onset hemorrhagic disease. Pediatr Radiol.

[8] Vestfrid MA, Oviedo AV (1975). Hemorragia de timo en el recién nacido, con especial referencia al hematoma. Rev Clin Esp.

[9] Walsh SV, Cooke R, Mortimer G, Loftus BG (1996). Massive thymic hemorrhage in a neonate: an entity revisited. J Pediatr Surg.

[10] Woolley MM, Isaacs H, Lindesmith G, Vollmer DM, Van Andelsberg S (1974). Spontaneous thymic hemorrhage in the neonate: report of two cases. J Pediatr Surg.

